# Prognostic Significance of Tumor–Stroma Ratio (TSR) in Head and Neck Squamous Cell Carcinoma: Systematic Review and Meta-Analysis

**DOI:** 10.3390/cells13211772

**Published:** 2024-10-26

**Authors:** Ilaria Girolami, Domenico Damiani, Rosa Negro, Monir Abousiam, Luca Gazzini, Luca Calabrese, Esther Hanspeter

**Affiliations:** 1 Department of Pathology, Provincial Hospital of Bolzano (SABES-ASDAA), Lehrkrankenhaus der Paracelsus Medizinischen Privatuniversität, via Lorenz Böhler, 5, 39100 Bolzano-Bozen, Italy; 2 Department of Otolaryngology, Provincial Hospital of Bolzano (SABES-ASDAA), Lehrkrankenhaus der Paracelsus Medizinischen Privatuniversität, 39100 Bolzano-Bozen, Italy

**Keywords:** tumor–stroma ratio, HNSCC, oral cancer, systematic review, meta-analysis, prognostic markers

## Abstract

The management of head and neck squamous cell carcinoma (HNSCC) relies heavily on TNM staging and WHO histologic grading; however, in recent years, the analysis of prognostic markers expressed in the tumor stroma has gained attention. The tumor–stroma ratio (TSR) quantifies the proportion of tumor tissue relative to the surrounding stromal tissue; it is assessed with the percentage of stromal tissue within the tumor area, with a cutoff point of 50% being widely used to discriminate high-stroma cancer. In this systematic review and meta-analysis, we investigated the potential prognostic role of the TSR in HNSCC. After a literature screening, 24 studies dealing with the TSR and survival outcomes were included. The TSR showed a significant association with overall survival (OS) in both unadjusted and adjusted measures (RR 2.04, CI 1.57–2.65, *p* < 0.01; HR 2.36 CI 1.89–2.94, *p* < 0.00001), with an even stronger prognostic potential in oral cavity/oral tongue cancers (RR 2.44 CI 1.84–3.22, *p* < 0.00001). The TSR also showed prognostic value when dealing with cancer-specific survival and was associated with a reduction in disease-free survival (DFS). In particular, the TSR also retained its prognostic role in terms of DFS when specifically considering early-stage cancers in both unadjusted and adjusted analyses (RR 1.81 CI 1.57–2.10, *p* < 0.00001; HR 2.09 CI 1.58–2.76, *p* < 0.00001). Therefore, we conclude that the TSR is a reliable prognostic marker that is easy to assess in routine histological slides and can be effectively implemented in the routine evaluation of HNSCC.

## 1. Introduction

Head and neck squamous cell carcinoma (HNSCC) constitutes a significant health challenge globally, with about one million new patients each year worldwide, hence being a relevant cause of mortality in some countries [1]. Unfortunately, approximately 60% of HNSCC cases are at an advanced stage (III-IV) at the time of diagnosis, which often correlates with a poor prognosis [2]. Traditionally, the management of these tumors relies heavily on TNM staging and histologic grading. However, these parameters may not always detect aggressive tumors, especially at an early stage (I-II) of HNSCC. Importantly, both TNM stage and WHO grade primarily consider the characteristics related to cancer, neglecting the features of the stroma [3].

In recent years, the evaluation of prognostic markers expressed in the tumor stroma has gained attention. Researchers have explored specific molecules within the stroma, such as alpha-smooth muscle actin, but these often require additional staining methods that are not routinely used in clinical practice [4]. An ideal prognostic marker should be assessable on routine hematoxylin and eosin (HE)-stained slides. The tumor–stroma ratio (TSR) quantifies the proportion of tumor tissue relative to the surrounding stromal tissue, and it is determined by assessing the percentage of stromal tissue within the tumor area. Using a microscope, with a low power lens (4× or 5×), the areas including a tumoral invasion front with the highest amount of stroma are selected; then, using a 10x lens, only the fields with both stroma and tumor on all sides of the field are assessed. Areas with necrosis, as well as major vascular structures and muscle tissue, are excluded [5]. A cutoff point of 50% is widely used in relevant studies to identify risk groups. Tumors are classified as either stroma-low (proportion of stroma < 50%, hence high TSR) or stroma-high (proportion of stroma ≥ 50%, hence low TSR) [5].

The more recent evidence in the literature indicates that tumor stroma plays a crucial role in cancer progression. Some cell types are found to be important, such as cancer-associated fibroblasts (CAFs), which can regulate cancer spread and influence metastasis through the production of extracellular matrix and growth factors [6]. Additionally, the tumor stroma is implicated in therapeutic resistance and also affects tumor biology in various solid tumors. Some studies have validated the prognostic value of the TSR in cancers beyond HNSCC, including colon, breast, gastric, and ovarian carcinomas [7,8,9,10]. The assessment of the TSR has promising prognostic value, as highlighted by seminal reviews on the topic, and can be deployed with minimal efforts in routine head and neck pathology.

In this systematic review and meta-analysis, we delve into the clinical significance of the TSR in HNSCC. By evaluating existing studies, we aim to clarify, with methodological strength, the potential of the TSR as a prognostic indicator and its practical implementation in routine head and neck pathology.

## 2. Materials and Methods

### 2.1. Literature Search

We aimed to follow the Preferred Reporting Items for Systematic Reviews and Meta-Analysis (PRISMA) guidelines [11]. The electronic databases MEDLINE-Pubmed and EMBASE were queried for items up until 24 March 2024. The search strategy was composed of free text terms referring to the tumor–stroma ratio and HNSCC, which were adequately combined but with no other restriction, to keep the retrieval as broad as possible. The complete search strategy for the two databases is found in Supplementary Material Table S1. There was no registration of a protocol (full PRISMA Checklist in Supplementary Material). We recognize that registering a protocol is a good practice, yet not mandatory nor a guarantee of the completion of a review; indeed, we checked on PROSPERO and found some protocols on the same topic, but with differences such as the specific population of HNSCC limited to oral cancer or different outcome measures (only adjusted or only odds ratios); moreover, none of these records are currently updated, nor are there corresponding reviews published to date. We fully acknowledge, however, that not registering a protocol is a limitation of our study; however, given that the published systematic reviews on the topic are methodologically different from our work, as they missed many relevant studies and did not analyze for unadjusted measures of outcome, we believe that there is no significant risk of the duplication of results nor that our work is biased by others.

### 2.2. Article Screening

The Rayyan QCRI reference manager web application [12] was used to help screen titles and abstracts. The full texts of the articles that fulfilled the initial screening criteria were acquired and reviewed for subsequent inclusion. The inclusion criteria were as follows: (1) a prospective or retrospective study design; (2) the presence of a comparison of prognosis between low TSR/stroma-high cases and high TSR/stroma-low cases; (3) the diagnosis of conventional HNSCC; (4) the presence of data about mortality for all causes, mortality for cancer, and the recurrence of disease; and (5) publication in a peer-reviewed journal or as a published abstract. The exclusion criteria were as follows: studies not concerning HNSCC, studies on animal models, studies not using morphological investigations, and studies not reporting on prognostic parameters. No language restrictions were applied, and the presence of an abstract only was not considered an exclusion reason.

### 2.3. Data Extraction

A standardized spreadsheet for extraction was used. The following data were extracted: author and publication year, country, type of paper, design of study if prospective or retrospective, number of cases, sites of cancer in the head and neck region, other markers investigated (morphological, immunohistochemical, and molecular), gender, age, pathologic TNM stage, tumor grading, cutoff (50% vs. other), type of specimen (biopsy vs. resection specimen), number of adjustments in survival analyses, and duration of follow-up. The primary outcomes were the number of deaths for cancer (disease-related survival—DSS) and for all causes (overall survival—OS), as well as the number of recurrences (disease-free survival—DFS) in patients with low TSR/stroma-high vs. high TSR/stroma-low. The secondary outcome was the hazard ratio, adjusted for the maximum number of confounders available, regarding the same outcome, taking those with high TSR/stroma-low as reference.

### 2.4. Quality Assessment

To evaluate the quality of studies, the Newcastle–Ottawa Scale (NOS) was used [13]. The NOS is an adequate tool for the quality assessment of observational studies and comprises three domains—the selection of participants, comparability, and outcomes. For the item of ascertainment of exposure, a point was given when the study used a resection specimen to establish the TSR. For the control item, studies that controlled their survival analyses for at least two confounders received one point, whereas studies that assessed the correlation with other clinical–pathological features received an additional point. All studies were scored for methodological quality with up to 9 points, with a score of ≤5 (out of 9) indicating a high risk of bias.

After quantitative evaluation, a modified version of the GRADE criteria [14] was used to evaluate the quality of evidence. The level of quality of evidence was downgraded for each of the following reasons: inconsistency of results by visual inspection of forest plot (more than 30% of studies with opposite results), more than 25% of studies at high risk of bias (defined as less than five points on NOS score), imprecision (number of events < 300), and publication bias (Egger’s test with *p* < 0.10). We did not evaluate indirectness, as this study concentrated on the specific population of HNSCC.

### 2.5. Quantitative Synthesis and Statistical Analysis

The open-source software R 4.4.0 (R Foundation for Statistical Computing, Vienna, Austria) with RStudio environment (RStudio Inc, Boston, MA, USA) and Review Manager 5.3 (The Nordic Cochrane Center, Cochrane Collaboration, Copenhagen, 2014) were used for analyses. For primary outcomes, pooled risk ratios (RRs) and 95% confidence intervals (CIs) of the risk of mortality and recurrence between low TSR/stroma-high and high TSR/stroma-low were calculated using DerSimonian–Laird random effects models [15]. For secondary outcomes, pooled hazard ratios (HRs) with 95% CIs adjusted for the maximum number of covariates available were also calculated to provide additional information if the relationship between the TSR and survival was influenced by potential confounders. The I2 metric and chi-square statistics [16] were used to assess heterogeneity across studies. Pooled estimates were also recalculated after a meta-regression analysis according to potential moderators at the study level. For publication bias, a visual inspection of the funnel plot and a formal Egger test were performed [17].

## 3. Results

### 3.1. Literature Search

**Figure 1:**
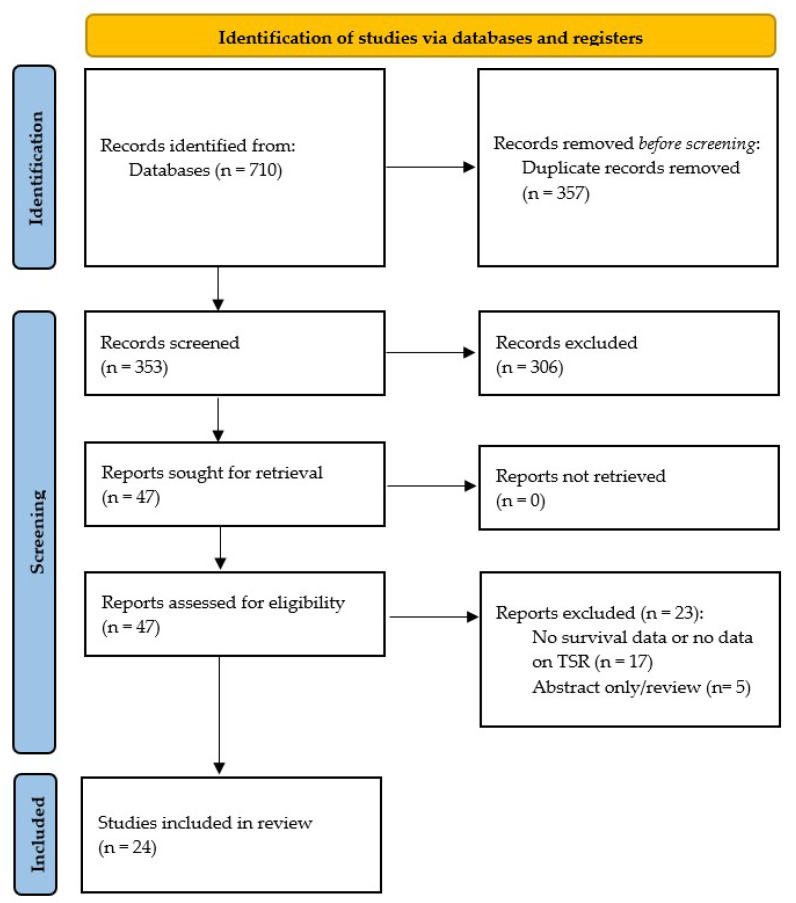
PRISMA flowchart of article screening.

There was a total of 353 articles identified after the removal of duplicates. Of these, 47 were considered as being potentially relevant after an initial title and abstract screening. After reading the full text, 24 studies were included. A detailed flow diagram of the literature screening process according to the PRISMA statement is shown in Figure 1.

### 3.2. Characteristics of Included Studies and Patients

The study consisted of 24 full-text articles published in the period 2014–2024. The geographic distribution of studies was as follows: Asia (n = 11) [18,19,20,21,22,23,24,25,26,27,28], Europe (n = 10) [29,30,31,32,33,34,35,36,37,38], South America (n = 2) [39,40], and Africa (n = 1) [41]. These studies involved a total of 3407 patients, of which 1457 (42.8%, raw unweighted proportion) demonstrated low TSR/stroma-high cancer. The majority of studies dealt with oral tongue/oral cavity cancer (n = 16); other studies dealt with different subsites, as follows: larynx (n = 4) [27,29,30,37], larynx and hypopharynx (n = 1) [35], lip mucosa and skin together (n = 1) [33], oropharynx (n = 1) [32], and nasopharynx (n = 1) [20]. The characteristics of the included studies are summarized in the Supplementary Material (Table S2).

### 3.3. Quality Assessment

The quality appraisal for the included studies is summarized in Supplementary Table S3. There were no studies with a high risk of bias, with a mean NOS score of 8. All selected studies reported adequate information on the selection of participants; a total of 16 studies had a shorter follow-up time (<60 months) and 8 studies reported only unadjusted measures of survival.

### 3.4. Association between TSR and Survival

**Figure 2:**
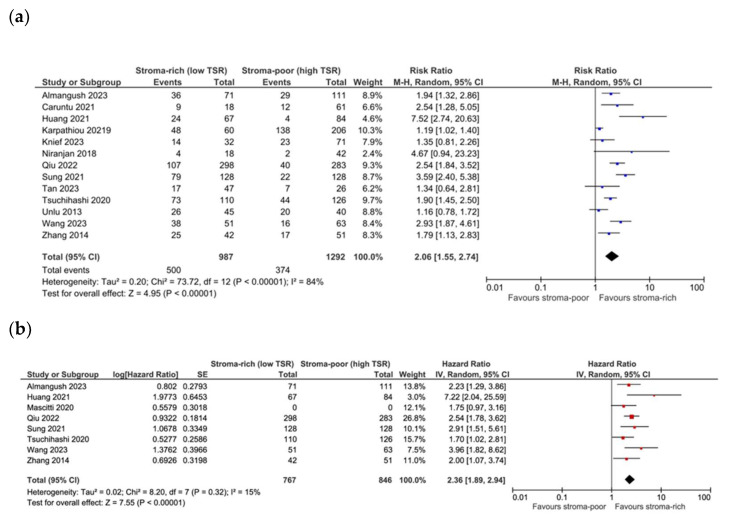
Forest plot of OS for unadjusted (**a**) [18,20,21,22,24,25,26,27,28,32,33,35,36] and adjusted (**b**) [18,20,22,24,26,28,32,38] measures.

**Figure 3:**
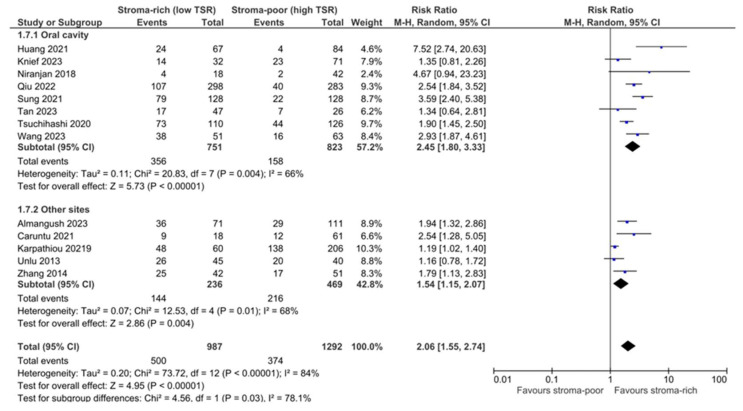
Forest plot of comparison of OS in oral cavity cancer vs. other subsites. Refs. [18,20,21,22,24,25,26,27,28,32,33,35,36].

The pooling data from 13 studies showed a significant association between low TSR/stroma-high and OS (RR 2.04, CI 1.57–2.65, *p* < 0.01, I^2^ 80%, τ^2^ 0.16). When pooling the data from eight studies that reported adjusted HRs, the association of a low TSR with reduced OS was stronger (HR 2.36 CI 1.89–2.94, *p* < 0.00001, I^2^ 15%, τ^2^ 0.02) and demonstrated a lower heterogeneity (Figure 2a,b). Egger’s test showed the potential occurrence of publication bias (*p* = 0.05), with the potential missing of studies with conflicting results. A meta-regression analysis according to the subsite of head and neck cancer (oral cavity vs. other) was performed and showed that the subsite explained up to 48% of the heterogeneity, with a significantly stronger association (*p* = 0.03) between a low TSR and worse OS in oral cavity cancer (RR 2.44 CI 1.84–3.22, *p* < 0.00001, I^2^ 66%, τ^2^ 0.11) compared to other subsites (RR 1.55 CI 1.14–2.11, *p* = 0.004, I^2^ 68%, τ^2^ 0.07) (Figure 3). In contrast, no significant difference was detected when subgrouping studies with adjusted HR data (oral cavity pooled HR 2.49 CI 1.83–3.39, *p* < 0.00001, I^2^ 36%, τ^2^ 0.05; other subsites HR 2.13 CI 1.41–3.21, *p* = 0.0003, I^2^ 0%, τ^2^ 0.00). The overall quality of evidence was moderate for potential publication bias. Focusing on studies dealing with early-stage cancer specifically, only two studies reported on OS, and no significant association between low TSR/stroma-high and OS was found (plot not shown).

**Figure 4:**
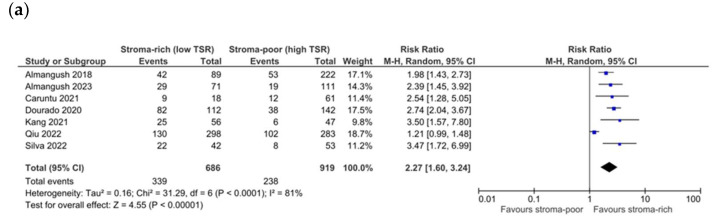

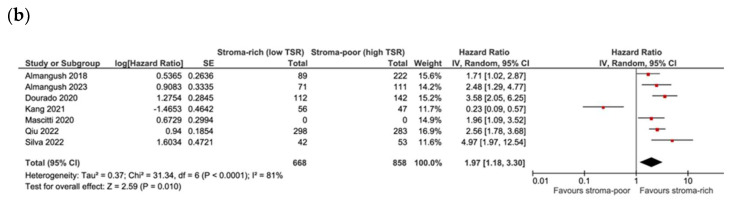
Forest plot of DSS for unadjusted (**a**) [19,22,31,32,33,39,40] and adjusted (**b**) [19,22,31,32,38,39,40] measures.

Seven studies reported on DSS, and when pooling data, a low TSR showed a significant association with mortality for cancer both in unadjusted and adjusted analyses (RR 2.27 CI 1.60–3.24, *p* < 0.01, I^2^ 81%, τ^2^ 0.16; HR 1.97 CI 1.18–3.30, *p* = 0.01, I^2^ 81%, τ^2^ 0.37) (Figure 4a,b). No significant differences were found when subgrouping according to subsite in both analyses (oral cavity, RR 2.24 CI 1.43–3.49, *p* = 0.0004, I^2^ 86%, τ^2^ 0.20 and HR 1.89 CI 1.04–3.44, *p* = 0.04, I^2^ 84%, τ^2^ 0.45; other subsites RR 2.44 CI 1.63–3.64, *p* < 0.00001, I^2^ 0%, τ^2^ 0.00 and HR 2.48 CI 1.29–4.77, *p* = 0.006, heterogeneity not applicable), but in both instances, a subgroup was composed only of one or two studies. Similarly, the Egger test revealed the potential for publication bias (*p* = 0.05). The overall quality of evidence was moderate for potential publication bias. Focusing on studies dealing with early-stage cancer only, three studies reported on DSS, and a low TSR showed a significant association with mortality for cancer in unadjusted analyses but not in adjusted analyses (RR 2.83 CI 1.72–4.67, *p* < 0.0001, I2 63%, τ2 0.12; HR 2.71 CI 0.96–7.65, *p* = 0.06, I2 74%, τ2 0.42). The quality of evidence was graded as low for imprecision (lower number of events) and potential publication bias.

### 3.5. Association between TSR and Recurrence

**Figure 5:**
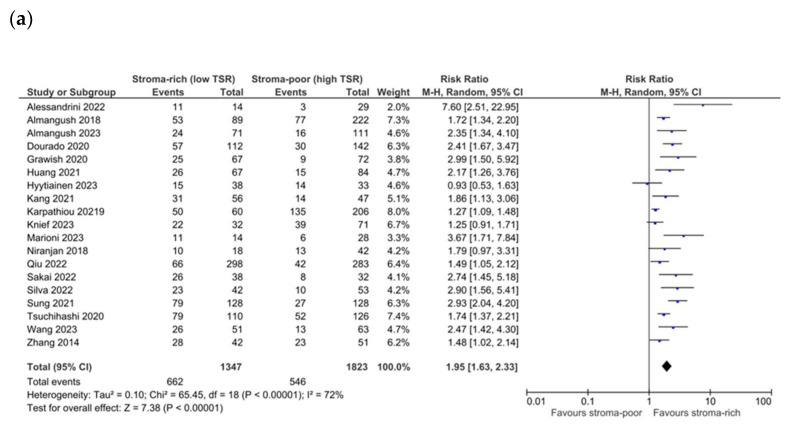

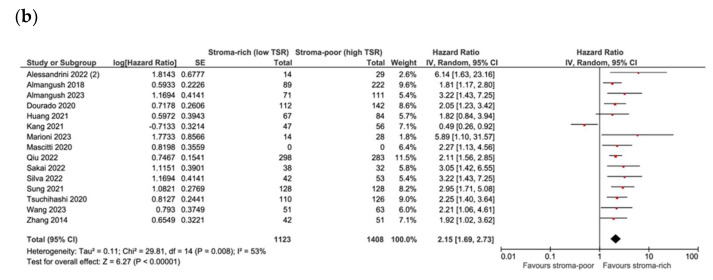
Forest plot of DFS for unadjusted (**a**) [18,19,20,21,22,23,24,26,28,30,31,32,34,35,36,37,39,40,41] and adjusted (**b**) [18,19,20,22,23,24,26,28,29,31,32,37,38,39,40] measures.

**Figure 6:**
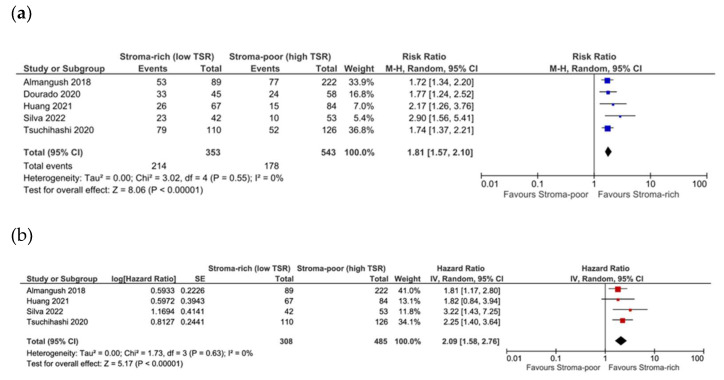
Forest plot of DFS for early-stage cancers for unadjusted (**a**) [18,26,31,39,40] and adjusted (**b**) [18,26,31,40] measures.

The pooling of data from 19 studies showed a significant association between low TSR/stroma-high and reduced DFS (RR 1.94, CI 1.64–2.29, *p* < 0.01, I^2^ 69%, τ^2^ 0.08). When pooling the data from 15 studies that reported adjusted HRs, the association of a low TSR with reduced DFS was stronger (HR 2.15 CI 1.69–2.73, *p* < 0.00001, I^2^ 53%, τ^2^ 0.11) (Figure 5a,b). Similarly to other analyses, the Egger test showed the potential occurrence of publication bias (*p* = 0.01), with the potential missing of studies with conflicting results. No significant differences were found when subgrouping according to subsite in both analyses (oral cavity, RR 1.92 CI 1.62–2.28, *p* < 0.00001, I^2^ 56%, τ^2^ 0.05 and HR 2.00 CI 1.53–2.61, *p* < 0.00001, I^2^ 59%, τ^2^ 0.11; other subsites RR 2.22 CI 1.33–3.73, *p* = 0.002, I^2^ 84%, τ^2^ 0.26 and HR 2.93 CI 1.75–4.90, *p* < 0.00001, I^2^ 16%, τ^2^ 0.05; test for subgroup analysis *p* = 0.60 and *p* = 0.20). The overall quality of evidence was moderate for potential publication bias. Focusing on studies dealing with early-stage cancer only, five studies reported on DFS, and a low TSR showed a significant association with recurrence both in unadjusted and adjusted analyses (RR 1.81 CI 1.57–2.10, *p* < 0.00001, I2 0%, τ^2^ 0.00; HR 2.09 CI 1.58–2.76, *p* < 0.00001, I2 0%, τ^2^ 0.00) (Figure 6a,b). The quality of evidence was graded as low for imprecision (lower number of events) and potential publication bias.

## 4. Discussion

In recent years, evidence has been accumulating that tumor stroma can influence the clinical behavior of solid tumors, while the stratification of tumors for the identification of cases at a high risk of poor outcome is still mainly based on TNM staging and WHO grading. However, both criteria rely only on the characteristics of the tumor, not taking into account the interaction of the tumor with the surrounding stroma. The important role of tumor stroma is documented in other cancer types such as colon, gastric, and breast cancer, and several studies have also highlighted its potential impact in HNSCC. The tumor–stroma ratio, defined as the proportion of stromal tissue within the tumor area with a commonly used cutoff of 50%, can be assessed in routine histological slides and has shown a promising capacity to identify cancers at a high risk of poor outcome.

In our systematic review with meta-analysis, we showed that a low TSR (hence a high stroma content) is strongly associated with worse survival outcomes in both adjusted and unadjusted analyses, with an overall moderate quality of evidence. This is in line with what was found out in previous systematic reviews on the topic [3,42]; however, the numerosity of studies was very low and the quality of evidence was low. Our stronger evidence highlights the relevant association of a low TSR with worse survival, thus emphasizing the role of this easily deployable marker in stratifying patients and defining the risk of poor outcomes. Indeed, the assessment of the TSR does not require anything other than routine H&E slides and has been shown to retain a high level of agreement among observers in studies on head and neck cancer [20,21,31,38,39]. The robustness of this marker and its independence from the type of specimen, whether a biopsy or surgical resection, has been investigated in dedicated studies that have reported a high level of concordance in the assessment between biopsy and resection specimens, also with the retention of prognostic value, when assessed in small biopsy material [29,30,37].

Moreover, almost all of the studies report the application of the method of van Pelt et al. [5] for the assessment of the TSR with the aim of standardization, and this adds value to the results of the review in terms of the quality of evidence, given that the heterogeneity at the study level is reduced. There were few studies proposing more than one cutoff or using whole-slide imaging (WSI) with the automated calculation of the TSR [24], but also in this occurrence, there were dichotomization in low and high proportions of stroma. Moreover, the strong association of the TSR with worse outcomes could also be explained considering the correlation between a high amount of tumor stroma and other features of cancer aggressiveness, such as perineural invasion, depth of infiltration, and treatment resistance, as has emerged from studies on clinical–pathological correlation [20,21,31,35]. This could have an even higher impact on early-stage cancers, where the identification of cases at high risk could offer the possibility of a more tailored and intensified treatment for survival improvement. Some studies dealt specifically with early-stage cancers reporting relevant association of the TSR with worse outcome [18,26,31,39,40], and the pooled estimate of both adjusted and unadjusted measures point towards a significant prognostic value of the TSR, specifically in early-stage cancers. Additionally, the prognostic value of low TSR/high stroma was even stronger concerning mortality for all causes in oral cavity cancer. Most of the studies included dealt with oral cavity/oral tongue cancer, but a significantly stronger association was found for unadjusted measures compared with the other head and neck subsites. Given that a relevant quota of oral cavity and, in particular, oral tongue cancers are amenable to surgically more conservative treatments, it could be of growing importance to have an additional reliable marker to identify cases at higher risk. Obviously, the TSR is still a “general” histological marker, and more specific markers of tumor stroma activity—such as different types of CAFs, as in one of the included studies [22], or even metalloproteinases (MMPs), which are known to have a role in the detachment of tumor cells and in the constitution of a stromal milieu favorable to tumor spreading—could have a prognostic value and refine the evaluation of the behavior of that specific tumor. Additionally, when dealing with tumor stroma, the complex inflammatory and immune landscape must also be taken into account. Tumor-infiltrating lymphocytes (TILs) have been investigated in many cancer types and have also been shown to have a prognostic value in HNSCC both alone and in combination with cancer grading [40]. However, it emerges from the literature that specific types of lymphocytes characterized by specific markers are related to different responses to therapy and different outcomes [43,44,45], while a direct correlation of TILs, both in general or specific cell types, with the TSR has not been fully elucidated. It could be speculated again that it is not the TILs population in general, defined as the ratio between tumor stroma and immune infiltrate [46], to have a prognostic value, but the combination of specific types of lymphocytes and specific types of stroma cells that defines the spreading potential and aggressiveness of cancer.

Finally, in our work, we also evaluated the quality of evidence, reporting an overall moderate quality of evidence according to GRADE, with downgrading to low only being required for the subset of studies dealing with early-stage cancers, as optimal information size in terms of number of events is not reached. We generally did not have indirectness nor imprecision, but we have to consider the fact that potential publication bias according to the Egger test was found, implying that some studies with contrasting results may be present. This, however, reinforces the need for additional experimental studies on homogeneous cohorts of patients to better refine the impact of the tumor–stroma ratio in HNSCC.

Our study has some limitations, which are mainly related to the included studies themselves. This is also linked to the future directions for research that our systematic review could suggest. Indeed, as assessed in all included studies, the TSR is a two-dimensional parameter defined on histological slides, while the tumor–stroma interaction is, in real life, a three-dimensional object. Spatial transcriptomics and spatial metabolomics are the cutting-edge technologies in the field of complex tumor microenvironment research and allow for the precise discrimination of markers and signatures related to aggressiveness, progression, and response to therapy [47]. Additionally, these analyses have also helped reveal important factors in tumorigenesis related to oral microbiota and its interaction with host and cancer tissues, with potential implications for cancer prevention both in primary and secondary prevention [48]. Moreover, these approaches are often non-invasive and non-destructive for tissues, hence allowing for the preservation of the possibility to have routine H&E slides and additional data from transcriptome analysis [49]. Notably, none of the included studies explored the correlation of the histological TSR with spatial transcriptomics data, and we believe that in the future, the combination of traditional histology, at best standardized as in most included studies, and new non-invasive technologies of spatial transcriptomics and metabolomics will reveal more precise relationships among markers at the single-cell level, microbiome elements, and cancer behavior. From this perspective, the histological TSR could serve as an initial evaluation step to be included in the routine assessment of HNSCC and to guide the subsequent analysis of cancer with more in-depth technologies to better stratify patients according to their risk of progression and recurrence and to their potential response to targeted therapy, thus realizing the promise of precision oncology.

## 5. Conclusions

The tumor–stroma ratio is an easily deployable marker that is usually assessed in routine H&E slides with good reproducibility and has shown promising prognostic value in many cancers. In this systematic review with meta-analysis, we demonstrated, with a rigorous methodology, that a high content of tumor stroma is strongly associated with worse overall survival, worse cancer-specific survival, and increased recurrence in head and neck cancer, both in early- and advanced-stage cancers. Even though more studies are still likely to refine the potential of this marker in specific settings, also in combination with transcriptomic analysis, the TSR appears to be reliable enough to be included in routine assessment of HNSCC.

## Data Availability

Data are contained within the article or Supplementary Material.

## Supplementary Materials

The following supporting information can be downloaded at: https://www.mdpi.com/article/10.3390/cells13211772/s1, Table S1: Search strategies; Table S2: Summary of included studies; Table S3: Methodological quality of cohort studies included in the meta-analysis.
