# Supplementary material for: Prognostic Significance of Tumor–Stroma Ratio (TSR) in Head and Neck Squamous Cell Carcinoma: Systematic Review and Meta-Analysis
Source: Cells. 2024 Oct 26;13(21):1772. doi: 10.3390/cells13211772 (PMC11545263; doi:10.3390/cells13211772)
Supplement: Supplementary file 1 [file cells-13-01772-s001.zip › Table S1 - searches.pdf]

Table S1. Search strategies.

Database	Search strategy
**Pubmed**	#1 tumor AND stroma AND ratio<br>#2 squamous<br>#3 #1 AND #2
**Embase**	#1 ('tumor'/exp OR tumor) AND ('stroma'/exp OR stroma) AND ('ratio'/exp OR ratio)<br>#2 squamous<br>#3 #1 AND #2<br>#4 #3 AND [embase]/lim NOT ([embase]/lim AND [medline]/lim)
