# Supplementary material for: Prognostic Significance of Tumor–Stroma Ratio (TSR) in Head and Neck Squamous Cell Carcinoma: Systematic Review and Meta-Analysis
Source: Cells. 2024 Oct 26;13(21):1772. doi: 10.3390/cells13211772 (PMC11545263; doi:10.3390/cells13211772)
Supplement: Supplementary file 1 [file cells-13-01772-s001.zip › Table S2 - studies.pdf]

Table S2. Summary of included studies.

				TSR low <50% (stroma-rich)					TSR high >50% (stroma-poor)							
**Author, year (Country)**	**Site**	**Exclusion criteria**	**Other features**	**N participants**	**N female**	**Age mean**	**TNM**	**Grade**	**N participants**	**N female**	**Age mean**	**TNM**	**Grade**	**Modality of evaluation**	**N adjustments**	**FU time median**
**Alessandrini, 2022 (Italy)*2 paper**	Larynx	NA	PD-L1, CD31, CD105 IHC	14	NA	NA	NA	NA	29	NA	NA	NA	NA	HE on biopsy and surgical resections	None	80
**Almangush, 2018 (Finland)**	Oral tongue	NA	Budding	89	46%	NA	T1 32.6%; T2 67.4%	G1 28.1; G2 44.9; G3 27%	222	47.3%	NA	T1 42.8; T2 57.2%	G1 36%; G2 41%; G3 23%	HE on surgical resections	5	NA
**Almangush, 2023 (Finland)**	Oropharynx	Concurrent HN cancers, palliative treatment, earlier treatments for HN cancer, histology other than SCC, or tissues not available	HPV, p16	71	21.1%	NA	T1 8.5%; T2 38%; T3 25.4%; T4 28.2%	G1 5.6%; G2 47.9%; G3 46.5%	111	24.3%	NA	T1 26.1%; T2 36.9%; T3 19.8%; T4 17.1%	G1 9.9%; G2 32.4%; G3 57.7%	HE on surgical resections	4	54
**Caruntu, 2021 (Romania)**	Oral lip mucos		Budding, necrosis, TILs	18	NA	NA	NA	NA	61	NA	NA	NA	NA	HE on surgical resections	NA	37

	a and skin														
**Dourado, 2020 (Brazil)**	Oral cavity	NA	Budding, margins	112	27.7%	NA	NA	G1 28.6; G2 58.9; G3 12.5%	142	24.6%	NA	NA	G1 28.2; G2 62%; G3 9.8%	HE on surgical resections	8	47
**Grawish, 2020 (Egypt)**	Oral cavity	NA	NA	67	32.8%	54	T1-2 10.5; T3-4 89.5%	G1 28.4%; G2 44.8%; G3 26.8%	72	34.7%	69	T1-2 66.7%; T3-T4 33.3%	G1 34.7%; G2 43.1%; G3 22.2%	HE on surgical resections	NA	>36
**Huang, 2021 (Korea)**	Oral cavity	Advanced tumors T3-T4, previous neoadjuvant therapy, no available data	NA	67	32.8%	NA	T1 31.3; T2 68.7%	G1 37.3%; G2-3 62.7%	84	31%	NA	T1 35.7%; T2 64.3%	G1 50%; G2-3 50%	Digitized HE resections	4	44,2
**Hyytiainen, 2023 (Finland)**	Oral tongue	No available histology or data	Budding, TILs, DOI, HEV, OCT3-4, HIFalpha	38	NA	NA	NA	NA	33	NA	NA	NA	NA	HE on surgical resections	NA	39
**Kang, 2021 (China)**	Oral tongue	No prior CHT-RT, no available data	Budding	56	57.1%	NA	T1-2 80.4%; T3-4 19.6%	G1 7.1%; G2 87.5%; G3 5.4%	47	51.1%	NA	T1-2 89.4%; T3-4 10.6%	G1 27.7%; G2 70.2%; G3 2.1%	HE on surgical resections	7	85

**Karpathiou, 2018 (France)**	Larynx and hypoharynx	Bx only, no available data	Budding, TILs, cell nest size, typ of stroma	60	NA	NA	NA	NA	206	NA	NA	NA	NA	HE on surgical resections	NA	NA
**Knief, 2024 (Germany)**	Oral cavity	No available data	None	32	34.4%	NA	T1 43.8%; T2 40.6%; T3 21.9%; T4 0%	G1 6.3%; G2 68.8%; G3 31.3%	71	31%	NA	T1 43.7%; T2 31%; T3 22.5%; T4 4.2%	G1 9.9%; G2 63.4%; G3 29.6%	HE on surgical resections	NA	57,7
**Marioni, 2023 (Italy)**	Larynx	NA	Budding, TILs, growth pattern, cell nest size	14	NA	NA	NA	NA	28	NA	NA	NA	NA	HE on biopsy and surgical resections	11	>60
**Mascitti, 2020 (Italy)**	Oral tongue	No available data, HPV pos, previous treatment	None	NA	NA	NA	NA	NA	NA	NA	NA	NA	NA	HE on biopsy and surgical resections	4	>36
**Niranjan, 2018 (India)**	Oral cavity	Previous tratment, mtx, other cancer, no available data	None	18	5.6%	NA	NA	G1 61.1%; G2 38.9%	42	14.3%	NA		G1 69%; G2 31%	HE on surgical resections	13	36

**Qiu, 2022 (China)**	Oral cavity	No data available, previous treatment, mtx, other cancer	CAFs	298	39.3%	NA	T1 28.5%; T2 50.3%; T3 12.8%; T4 8.4%	G1 31.9%; G2 54.4%; G3 13.8%	283	36.4%	NA	T1 32.5%; T2 51.6%; T3 9.9%; T4 6%	G1 42.8%; G2 45.2%; G3 12%	HE on surgical resections	5	30,3
**Sakai, 2022 (Japan)**	Oral tongue	NA	Buddin g	38	NA	NA	T1 23.7%; T2 47.4%; T3 28.9%	NA	32	NA	NA	T1 50%; T2 34.4%; T3 15.6%	NA	HE on surgical resections	10	47
**Silva, 2022 (Brazil)**	Oral cavity	Advanced cancer	Buddin g, TILs	42	NA	NA	NA	NA	53	NA	NA	NA	NA	HE on surgical resections	3	60
**Sung, 2021 (South Korea)**	Oral cavity	NA	Buddin g, TILs	128	NA	NA	NA	NA	128	NA	NA	NA	NA	HE and IHC automate d assessme nt on surgical resections	5	66
**Tan, 2023 (Turkey)**	Oral cavity	No available data	Buddin g, WPOI, TILs, stroma type, cell nest size	47	48.9%	NA	T1 27.7%; T2 44.7%; T3 19.1%; T4 8.5%	G1 8.5%; G2 83%; G3 8.5%	26	50%	NA	T1 34.6%; T2 50%; T3 7.7%; T4 7.7%	G1 26.9%; G2 61.5%; G3 11.5%	HE on surgical resections	5	30

**Tsuchihashi, 2020 (Japan)**	Oral cavity	NA	None	110	43.6%	NA	T1-2 64.5%; T3-4 33.6%	G1 45.5%; G2-3 52.7%	126	39.7%	NA	T1-2 91.3 %; T3-4 7.9%	G1 57.9%; G2-3 39.7%	HE on surgical resections with image analysis	5	NA
**Unlu, 2013 (Turkey)**	Larynx	NA	NA	45	6.7%	NA	T1 0%; T2 8.9%; T3 44.4%; T4 46.7%	G1 28.9%; G2 44.4%; G3 15.6%	40	2.5%	NA	T1 0%; T2 2.5%; T3 52.5%; T4 45%	G1 17.5%; G2 50%; G3 32.5%	HE on surgical resections	NA	48
**Wang, 2023 (China)**	Oral cavity	Previous treatment, HPV pos, DOI<2mm, no available data	NA	51	43.1%	NA	NA	G1 74.5%; G2-3 25.5%	63	36.5%	NA	NA	G1 74.6%; G2-3 25.4%	HE and IHC automated assessment on surgical resections	9	52
**Zhang, 2014 (China)**	Nasopharynx	NA	None	42	26.2%	45	T1 11.9%; T2 26.2%; T3 28.6%; T4 33.3%	NA	51	33.3%	48	T1 5.9%; T2 35.3%; T3 45.1%; T4 13.7%	NA	HE on surgical resections	8	67

Abbreviations: CAFs, cancer-associated fibroblasts; DOI, depth of invasion; IHC, immunohistochemistry; HE, hematoxylin & eosin; HN, head and neck; NA, not available; TILs, tumor-infiltrating lymphocytes; TNM, tumor-node-metastasis; TSR, tumor-stroma ratio; WPOI, worst pattern of invasion
