# Supplementary material for: Prognostic Significance of Tumor–Stroma Ratio (TSR) in Head and Neck Squamous Cell Carcinoma: Systematic Review and Meta-Analysis
Source: Cells. 2024 Oct 26;13(21):1772. doi: 10.3390/cells13211772 (PMC11545263; doi:10.3390/cells13211772)
Supplement: Supplementary file 1 [file cells-13-01772-s001.zip › Table S3 - quality.pdf]

Table S3. Methodological Quality of Cohort Studies Included in The Meta-Analysis*

First author, publication year	Representativeness of the exposed cohort	Selection of the unexposed cohort	Ascertainment of exposure†	Outcome of interest not present at start of study††	Control for important factor or additional factors†††	Assessment of outcome	Follow-up long enough for outcomes to occur††††	Adequacy of follow-up of cohorts	Total quality scores
Alessandrini, 2022§	*	*	*	*	*	*	*	*	8
Almangush, 2018	*	*	*	*	**	*	-	*	8
Almangush, 2023	*	*	*	*	**	*	-	*	8
Caruntu, 2021	*	*	*	*	*	*	-	*	7
Dourado, 2020	*	*	*	*	**	*	*	*	9
Grawish, 2020	*	*	*	*	*	*	-	*	7
Huang, 2021	*	*	*	*	**	*	-	*	8
Hyytiainen, 2023	*	*	*	*	**	*	-	*	8
Kang, 2021	*	*	*	*	**	*	*	*	9
Karpathiou, 2018	*	*	*	*	**	*	-	*	8
Knief, 2024	*	*	*	*	*	*	-	*	7
Marioni, 2023	*	*	*	*	**	*	*	*	9
Mascitti, 2020	*	*	*	*	**	*	-	*	8
Niranjan, 2018	*	*	*	*	*	*	-	*	7
Qiu, 2022	*	*	*	*	**	*	-	*	8
Sakai, 2022	*	*	*	*	**	*	-	*	8
Silva, 2022	*	*	*	*	**	*	*	*	9

Sung, 2021	*	*	*	*	**	*	*	*	9
Tan, 2023	*	*	*	*	**	*	-	*	8
Tsuchihashi, 2020	*	*	*	*	*	*	-	*	7
Unlu, 2013	*	*	*	*	*	*	-	*	7
Wang, 2023	*	*	*	*	**	*	-	*	8
Zhang, 2014	*	*	*	*	**	*	*	*	9

Original studies were analyzed in the quality assessment.

* A study could be awarded a maximum of one point for each item except for the item Control for important factor or additional factor. The definition/explanation of each column of the Newcastle-Ottawa Scale is available at http://www.ohri.ca/programs/clinical_epidemiology/oxford.htm.

§ The same cohort is used to assess two different outcome measures in two separate papers.

† For this index, one point was given if in Method section the TSR was assessed with on resection specimen.

†† Being outcome of interest mortality, we took as outcome of interest for assessment of quality if the overall survival or the recurrence rate was assessed.

††† A maximum of 2 points could be awarded for this item. Studies that controlled their survival analyses for at least two confounders received one point, whereas studies that assessed also the correlation with other clinic-pathological features, an additional point.

†††† A cohort study with a mean/median follow-up time ≥5 y (60 months) takes one point.
